# Annotations

**Published:** 1905-12-02

**Authors:** 


### ANNOTATIONS.

### The Royal Commission on the Poor Laws.

Since the Government announced their intention to appoint a Royal Commission to inquire into the working of the laws relating to the relief of poor persons in the United Kingdom, we have anxiously awaited the nomination of the actual members of the Commission. The practical value of any inquiry such as this, which is more than likely to form the basis of future legislation, must of necessity depend largely upon the scientific capacity and practical experience of those to whom the inquiry is entrusted. It cannot be said that the announcement on Wednesday of the personnel of the Commission is likely to give rise to much satisfaction to those whose calling brings them into close touch with the existing poor-laws, at least, so far as these bear upon the provision for the sick and destitute. For by a strange oversight there is but one medical man among the eighteen members of the Commission; and although three ladies have been nominated, not one of these is a trained nurse. These facts are to be lamented the more since some of the most urgent and pressing reforms needed in our present poor-laws have reference to the treatment of the sick. We have not space to enumerate here the startling deficiencies which exist in this field of public philanthropy; but these are numerous and startling enough, and it is not to be expected that their full significance will be appreciated by individuals, however circumspect and impartial they may be, who have had no practical experience upon which to found their judgments. It is to be deplored that in a country which is peculiarly rich in men of science, the tendency seems to be to overlook the counsel of such men in most practical matters of the State.

### A Great Medical Scientist.

There passed away, at Oxford, on Thursday night last week, and was buried on Tuesday, amid remarkable manifestations of regret, a great medical scientist who entirely merited the distinctions which he gained during a career extending over nearly fourscore years. Sir John Burdon-Sanderson, who was born at Newcastle-on-Tyne, and educated in the medical schools of Edinburgh and Paris, first came to the front as Medical Officer of Health for Paddington. He held this position for eleven years, being concurrently, for a portion of the time, on the staff of the Middlesex Hospital and the Brompton Hospital for Consumption. At both these institutions his services were warmly appreciated. The admirable manner in which he discharged the duties of medical officer resulted in his nomination, by the Local Government Board, to inquire into and report upon an outbreak of disease in Kent, which was classified for the first time as diphtheria. He subsequently conducted investigations into, and published reports upon, other infectious and contagious maladies. To him the community is largely indebted for the elucidation of the real character of cholera, as well as for much light upon the true nature and origin of tuberculosis. His researches at this period were not confined to the ills that afflict mankind; he devoted considerable attention to the diseases of animals, and his researches on the cattle plague were most illuminating. His appointment to the office of first Superintendent of the Brown Institution, which he held for seven years, was followed by his election to the Jodrell Professorship of Physiology in University College, London, and a few years later he was chosen, in spite of formidable opposition, to occupy the newly-established Waynflete Chair of Physiology at Oxford. In 1895 he succeeded Sir Henry Acland as Regius Professor of Medicine at the University, and did not resign it until last year. Age did not wither his infinite capacity, and even in the medical and scientific world, which delighted to do him honour, the full effect of the work he accomplished remains to be appraised. The anti-vivisectionists vehemently denounced him for the experiments which added to the sum of human knowledge, but they never called his disinterestedness in question, and bore him no personal enmity. His pupils are amongst the most eminent men of to-day, and his name will be held in veneration as that of a savant who certainly left the world richer than he found it.

### Opsonins and Immunity.

A Meeting of the Royal Medical and Chirurgical Society, held last Tuesday, is likely to remain a memorable one, for a good deal of light was focussed on the problem of producing artificial immunity, especially against tuberculosis. Dr. David Lawson and Dr. Ian Struthers Stewart communicated some highly instructive observations upon the variations which occur in the opsonic index of the blood at varying intervals after the injection of tuberculin (T.R.) In the first place they found that the opsonic index of the blood was materially higher in healthy individuals than in those affected with pulmonary tuberculosis. It was further observed that an injection of tuberculin was followed by a negative phase in which the opsonic index was reduced; but after a while a positive phase occurred and the opsonic index gradually rose again until it had reached a higher level than before the injection. A second injection of tuberculin during the negative phase, caused a still further fall in the opsonic index, and therefore might be considered as highly deleterious. On the other hand, a second injection given during the positive phase was successful, after the consecutive negative phase had passed off, in raising the opsonic index to a permanently higher level than before. This being so it seems reasonable to assume that repeated injections of tuberculin given at the proper time and with suitable regulations may be of the utmost value in the treatment of tuberculosis, provided always that the patient is not too ill to withstand the temporary depression of the negative phase. But to determine the opsonic power of the blood requires complicated apparatus and considerable skill. As soon as a simpler method has been devised, the use of tuberculin promises to become a routine method of treating tuberculous infection.

